# Knowledge development, technology and quality of experience in collaborative learning: a perspective from Saudi Arabia universities

**DOI:** 10.1007/s11135-022-01476-9

**Published:** 2022-08-23

**Authors:** Abdulrahman Alyami, Salvatore F. Pileggi, Igor Hawryszkiewycz

**Affiliations:** 1 School of Computer Science, Faculty of Engineering & IT, University of Technology Sydney, Sydney, Australia; 2 College of Computer and Information Sciences, Jouf University, Al-Jawf, Kingdom of Saudi Arabia

**Keywords:** Collaborative technology, Collaborative learning, Knowledge Development, Knowledge sharing

## Abstract

Technology has recently gained relevance within collaborative learning environments to provide robustness, agility and flexibility. Several recent studies have investigated the role of technology, as well as researchers have defined different metrics to assess learning outcomes and experience along the collaborative knowledge development process. More recently, technology has played a key role to face the new challenges related to COVID-19, which forced to move on remote or hybrid learning. This research focuses on the quality of learning experience in terms of academic performance and perceived satisfaction. From a methodological point of view, a conceptual framework has been proposed and a quantitative study has been conducted among undergraduate and postgraduate students that are undertaking programs related to System Design in Saudi Arabia universities. 152 responses have been collected through an online survey and analysed using SPSS and SmartPLS. Results show a positive impact of technology along the collaborative knowledge development process and a strong correlation among the different quality of learning experience parameters considered. Indeed, despite some challenges, an integrated use of technology seems to properly support the most pressing needs in terms of quality experience, while the well-known social/educational issues related to the COVID-19 pandemic are not object of this study. Those findings are expected to contribute to the Saudi Arabia’s vision 2030 and, more holistically, to the assessment of collaborative learning environments that extensively rely on technology.

## Introduction

Saudi Arabia’s vision 2030 (Government of Saudi Arabia, 2020) is a strategic plan established in 2016 by Crown Prince Mohammed bin Salman. The main purpose of the vision is to overcome the dependence on oil by expanding its economy on developing public services sectors (i.e., education, health, infrastructure, tourism, and recreation). As a part of the Saudi Vision 2030, the Ministry of Higher Education explicitly aims at improving e-learning environments which are expected to become more effective in practice (Government of Saudi Arabia, 2020).

More recently, the COVID-19 pandemic has forced the World to change and re-design many aspects of daily life and significant restrictions have been enforced by governments, such as closures of borders, social distancing, and lockdowns. Undoubtedly, Covid-19 has raised strong challenges also in education to maintain learning at the different levels (Adnan 2020). Educational organizations moved the facto from face-to-face to virtual learning environments, relying extensively on online resources (Qazi et al. 2021; Tawafak et al. 2021).

Focusing more specifically on the broad area of subjects related to system design, this research targets collaborative environments where students are requested to work collaboratively as a group to develop core tasks. Along the learning process, which also involves instructors who may provide feedback in different forms at key stages, students need to develop a common understanding of problems and goals that ultimately result in knowledge. Current technology contributes to establish a flexible and resilient collaborative learning environment to develop and share knowledge (Gokhale 1995; Pinheiro and Simões 2012; Recker et al. 2013; Resta and Laferrière 2007). The collaborative knowledge development process can be understood in multiple ways and, in the context of this work, is seen as the ability to employ data and information within groups to produce ideas by applying the target methods and techniques proposed (Recker et al. 2013). In general terms, knowledge sharing is considered the process to communicate, exchange and eventually enrich the developed knowledge (Baanqud et al. 2020). Several studies have reported the effectiveness of adopting collaborative technology in knowledge development & sharing and there are evidences of high performance within interactive environments (Gokhale 1995; Lipponen and Lallimo 2004; Pinheiro and Simões 2012; Recker et al. 2013).

Apart from the already mentioned practical relevance of the research conducted, at a more theoretical level there is a fundamental lack of study that explicitly addresses the relationship between traditional learning models (for instance modelled according to the Bloom’s taxonomy, which defines different levels of learning (Bloom 1984) and the process of knowledge development and sharing (defined for example by the Nonaka’s theory (Nonaka 1994) in collaborative learning environments. Additionally, the quality of learning experience is not always assessed as a whole looking at the different dimensions or aspects.

Given the large scope of the mentioned theories, this paper only focuses on the aspects that are most relevant for the extent and intent of the study conducted (e.g. socialization and externalization from Nonaka’s theory and application, analysis and creation from Bloom’s Taxonomy).

More concretely, this study aims to (i) assess the impact of collaborative technology on the overall quality of learning experience at different levels of learning, (ii) investigate the process of knowledge development & sharing within collaborative environments which rely extensively on technology to gain flexibility and resilience and (iii) understand the relationship between academic performance and perceived satisfaction in such learning environments.

This study adopts a quantitative method to conduct a research among undergraduate and graduate students involved in programs addressing some kind of system design in Saudi Arabia universities. An online survey has been designed to assess the collaborative knowledge development process within environments that extensively rely on technology and the related overall quality of experience.

The outcomes of this study are expected to contribute to some aspects of Saudi Vision 2030, which extensively depends on the effective adoption of technology. The recent experience during the COVID-19 pandemic has further increased the potential relevance of the study (Hassounah et al. 2020). The theoretical findings and their practical implications are expected to support further exploration for researchers and academics in learning contexts.

The paper follows with a focused discussion on the related work, then the research methodology is addressed and the proposed conceptual model and associated hypotheses are proposed. Finally, results are analysed and discussed as well as current limitations and future work.

## Related work

Many studies from literature have reported the effects of adopting technology into the educational process in different contexts and situations (Alyami et al. 2020; Baanqud et al. 2020; Recker et al. 2013; Su et al. 2010; Wang 2009). According to the mentioned studies reported the extensive adoption of technology within learning environments may affect the quality of learning experience, including final outcomes. Indeed, a proper use of technology can facilitate students to effectively acquire skills, experience, and knowledge. Additionally, the establishment of a consolidated and agile technological environment is likely to make the whole learning experience more enjoyable for students (Ruiz et al. 2021). There are different possible practical effects, for instance the study in (Schrader and Grassinger 2021) investigated the relationship among enjoyment and performance in attributional feedback.

On the other side, according to (Ruiz et al. 2021), in the pre-COVID period, online resources provide flexibility and enable a more effective learning process which improves students’ habits and facilitates the creation of skills through blended learning settings. Due to recent challenges – i.e. COVID-19 - Collaborative Technology (Adedoyin and Soykan 2020) plays a more and more important role in maintaining learning activities remotely. It is expected to fully support students around the world by providing a scalable environment to interact and collaborate remotely (Alenazy, Mugahed Al-Rahmi, & Khan, 2019; Hernández-Sellés, Pablo-César Muñoz-Carril, & González-Sanmamed, 2019; Qazi et al. 2021; Tawafak et al. 2021). However, despite the most modern technology it is supposed to be effective also in education, its impact on the quality of learning experience across the different disciplines and activities is still to be fully assessed.

Collaborative learning environments are recognised as a key factor to drive an effective and efficient developing of knowledge. For instance, in (Hmelo-Silver and Barrows 2008) the authors analysed how medical students can achieve knowledge building as a team supervised by an instructor in problem-based learning. The study reported in (Micari and Pazos 2021) addresses the social cognitive outputs among students organised in groups. In both mentioned cases (Hmelo-Silver and Barrows 2008; Micari and Pazos 2021), group work positively contributes to knowledge development & sharing. However, such studies do not fully address the impact of the extensive adoption of collaborative technology on the different aspects of the overall quality of learning experience, such as effectiveness, efficiency, enjoyment, eventually academic performance, and satisfaction.

Involving technology in collaborative learning has a tangible impact on academic performance (Resta and Laferrière 2007) by providing flexibility for engagement. The most commonly accepted assessment metrics for the quality of experience are performance and satisfaction (Al-Rahmi et al. 2014; Al-Rahmi and Zeki 2017; Alalwan et al. 2019; Tullis and Albert 2013). While performance assesses the learning outcome, satisfaction refers to the student’s perceived quality of experience. However, to assess an overall learning experience, it is important to consider fine-grained metrics, such as effectiveness (the capability to deliver the requested outcome) and efficiency (the capability to deliver an outcome with certain constraints, e.g., time) (Lin et al. 2020; Renner et al. 2014). Rarely, effectiveness, efficiency, and enjoyment are simultaneously considered to assess performance and satisfaction.

At a more theoretical level, we perceive a certain lack of analysis of the quality of experience in relationship with traditional learning dimensions/levels specified by Bloom’s Taxonomy (Bloom 1984). Bloom’s Taxonomy is a framework that presents different levels of learning. The levels can guide instructors in teaching, assessing, and understanding how to provide effective interactive learning environments. Additionally, activities to develop and expand knowledge as per Nonaka’s theory (Nonaka 1994) are not fully investigated.

## Methodology

This study adopts a quantitative method as suggested by relevant studies in literature. Based on the review of existing works, an integrated conceptual model including heterogeneous concepts has been proposed. A questionnaire has been designed to define and measure the relationships existing among target concepts. Such a questionnaire is graded according to a five-point Likert scale (Allen and Seaman 2007).

The data has been collected through an online survey conducted among undergraduate and postgraduate students undertaking programs related to System Design in Saudi Arabia universities (Jouf University, Imam Abdulrahman Bin Faisal University, and Jubail University College). The questions were proposed in both English and Arabic to facilitate their understanding. The data was collected during the pandemic period when universities were adopting remote learning.

A pilot study has been conducted on a small scale to consolidate and refine the proposed conceptual model. Finally, 152 responses have been collected and analysed. Such data has been analysed by using SPSS (George and Mallery 2018) and SmartPLS (Ringle et al. 2014). The former has been used for statistical analysis, while the latter provides Measurement Model Analysis and Structural Equation Modelling (Ringle et al. 2014).

## Conceptualisation and Hypotheses

This section proposes an overview of the conceptual model, which is discussed both with the associated hypotheses in context looking at existing theories.

### Conceptual model

**Fig. 1:**
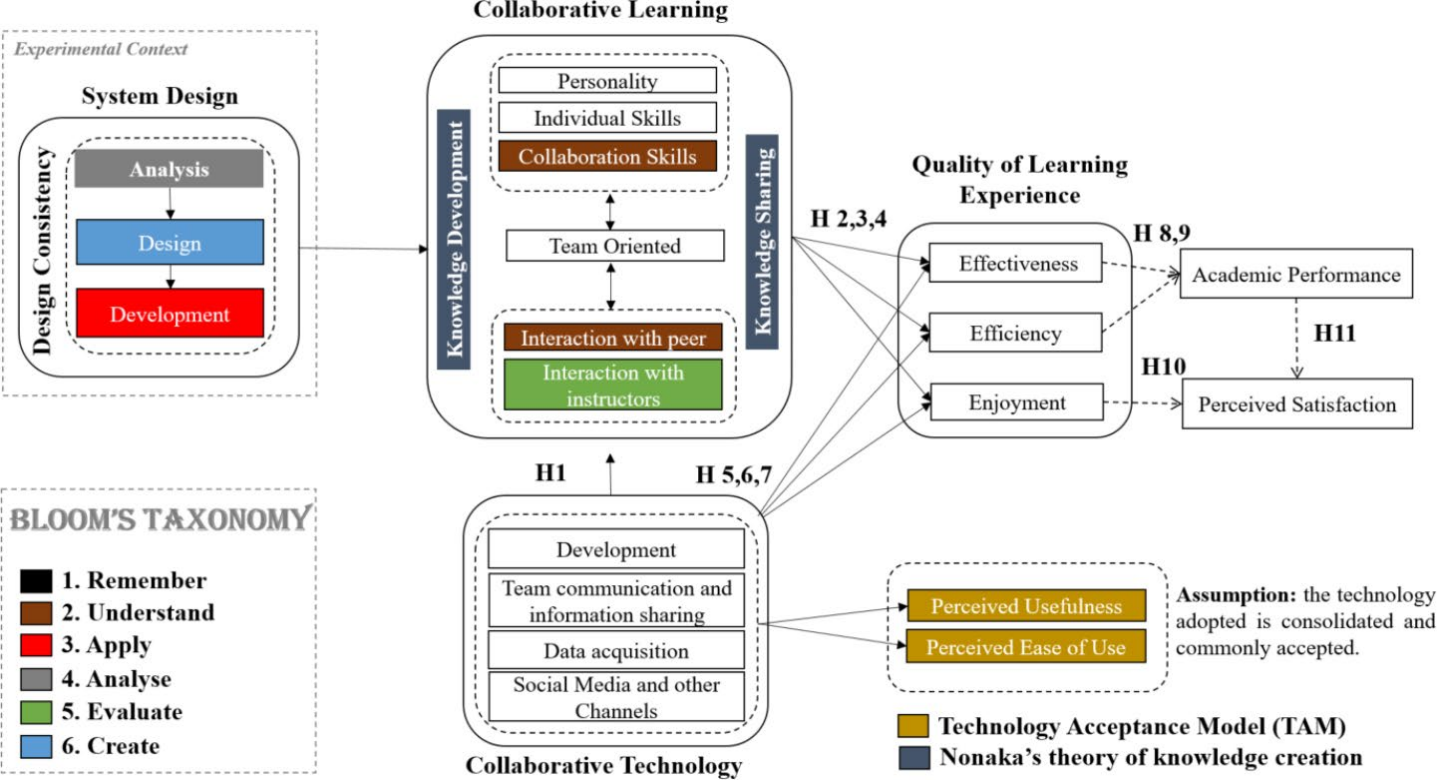
Conceptual model

The proposed conceptual model is depicted in Fig. 1. The experimental context of the study focuses explicitly on System Design (Hoffer et al. 2013). System Design is commonly understood as a set of processes that aim to the specification of the different system elements and components that meet a given set of requirements. More concretely, we put emphasis on the phases of analysis, design and development that can be associated respectively with analysis, creation and application in the Bloom’s taxonomy.

Within the model, we distinguish among independent and dependent concepts, where an independent concept has the potential to influence the dependent concept (Stewart 1978). The main independent concepts are related to Collaborative Technology (Alahmari 2019), while those concepts related to Collaborative Learning (Recker et al. 2013) and Quality of Learning Experience (Al-Rahmi et al. 2014; Alalwan et al. 2019; Lin et al. 2020; Renner et al. 2014) are understood as dependent concepts. Additionally, the framework assumes the process of collaborative learning associated with the knowledge development & sharing as in Nonaka’s theory. Nonaka’s conceptual approach addresses a set of principles for knowledge creation in a generic organizational context. Looking specifically at a collaborative environment in education, some principles and considerations may be reused in scope looking at the intrinsic need to develop knowledge in co-operation with others and to share it at different levels of learning. Finally, the Technology Acceptance Model (TAM) is considered as an assumption, meaning we are assuming collaborative technology as a consolidated and commonly accepted asset within the target community.

The next subsections describe more in detail the main blocks in the conceptual model.

#### Collaborative learning

Collaborative Learning refers to a learning process involving more than one student to achieve a common goal (Recker et al. 2013). There are very many factors, such as individual personality (Hernández-Sellés et al., 2019) and skills (Gomez et al. 2010), collaboration skills (Baber 2021), interaction among peers and instructors (Al-Rahmi et al. 2014; Habes et al. 2018; Qureshi et al. 2021), that can potentially affect collaborative learning to develop knowledge properly. Those fine-grained concepts are not explicit object of this study.

Collaborative learning environments have been object of investigation in several studies (Baanqud et al. 2020; Ghavifekr 2020; Hmelo-Silver and Barrows 2008; Recker et al. 2013; Su et al. 2010; Wang 2009); in general terms, group-work has normally a positive impact on the learning process and the consequent knowledge building within a given group. Knowledge can be defined in many different ways depending on the context. However, it is quite commonly associated with information, data, experience, expertise, fact, wisdom, and action (Akude 2014). Knowledge is the capability to use the information and data in practice (Applehans et al. 1999), while the authors in (Argyris, 1992) define knowledge as the ability for effective practical actions.

The relationship between collaborative learning and knowledge building has been further investigated in (Recker et al. 2013). It proposes an empirical study on post-graduate students to assess the impact of technology on the cognitive process within a group. Other studies (Baanqud et al. 2020; Su et al. 2010; Wang 2009) address more specific aspects.

In recent years, more and more works reiterate the relevance of knowledge development & sharing in education (Van Weert 2006) as well as collaboration is seen as a key factor to foster creativity and the consequent conversion of ideas into design. Last but not least, in (Ghavifekr 2020) the authors explicitly address the relationship between collaborative learning and academic performance.

As far as authors know, there is no model that explicitly related Bloom’s taxonomy to collaborative learning. The model proposed in this study assumes a partial mapping as in Fig. 1 with a focus on aspects associated with collaboration. However, learning environments could be more complex in fact, for instance assuming instructors as an active part of the knowledge building process.

#### Collaborative technology

Ideally, Collaborative Technology is expected to support a collaborative learning process along the different phases (Alahmari 2019). Technology plays a critical role in modern education (Peled et al. 2022). The proposed conceptual model assumes collaborative technology mainly aimed to content sharing, task development (Recker et al. 2013), communication (including Social Media (Al-Rahmi et al. 2015)) and data acquisition (Alahmari 2019; Habes et al. 2018). Those aspects have been proved to be key factors according to several studies. For instance (Recker et al. 2013) puts emphasis on the relevance of technology in task development, as well as (Al-Rahmi, Othman, Yusof, et al., 2015; Habes et al. 2018) address social media and (Krajcik et al. 1994; Marcu and Spiller 2020) deal with information sharing.

The adoption of social media is recognised as a valuable asset in learning activities (Alghizzawi et al. 2018). It has the potential to contribute to lead students beyond ideas and further discover through deepening (Al-Rahmi et al. 2015), as well as to better develop skills and experience (Al-Mohammadi and Derbel 2014).

Several studies addressed the relationship between technology adoption in learning and collaboration (Gan et al. 2015), problem-solving (Unal and Cakir 2021), even within specific learning contexts (e.g. languages (Kern 2006)). In general terms, those studies have observed an acceptance of technology and, therefore, its effectiveness in learning (Raja and Nagasubramani 2018).

#### Quality of learning experience

Quality of Learning Experience is a broad concept which in the context of this study is associated with effectiveness (Lin et al. 2020; Renner et al. 2014), efficiency (Renner et al. 2014), and enjoyment (Lin et al. 2020). Those three factors can reflect standard metrics such as academic performance and satisfaction (Al-Rahmi et al. 2014; Alalwan et al. 2019).

The study in (Lin et al. 2020) investigated the role of perceived enjoyment in a blended environment, where students, organised in teams, are expected to collaborate and engage in learning activities to increase their effectiveness. Additionally, the authors looked at students’ overall quality of experience, with an explicit focus on their perception. According to (Hsu and Lin 2008) consider enjoyment as a key factor for engagement which is further empowered by social media adoption. (Renner et al. 2014) reiterated the relevance of effectiveness and efficiency as essential assessment metrics. Despite their relevance, rarely effectiveness, efficiency and enjoyment are simultaneously considered to assess performance and satisfaction.

Students’ performance and satisfaction are often object of study. Technology Acceptance Model (TAM), constructivism (Vygotsky 2020), and communication theories (Jensen 2020; Walther 1996) have been adopted to assess the effects of social media use on academic performance (Alalwan et al. 2019). In (Al-Rahmi et al. 2014), authors observed the influence of social media on different driver factors, such as interaction, engagement, ease of use and usefulness.

### Hypotheses

The objective of this study is to assess the collaborative knowledge development & sharing process and the related overall quality of experience within environments that extensively rely on technology. The following hypotheses are associated with the underlying theory:

*H1: Collaborative technology contributes to knowledge development and sharing.*

*H2: Collaborative learning contributes to effectiveness.*

*H3: Collaborative learning contributes to efficiency.*

*H4: Collaborative learning contributes to enjoyment.*

*H5: Collaborative technology contributes to effectiveness.*

*H6: Collaborative technology contributes to efficiency.*

*H7: Collaborative technology contributes to enjoyment.*

*H8: Academic performance depends on effectiveness.*

*H9: Academic performance depends on efficiency.*

*H10: Perceived satisfaction depends on enjoyment.*

*H11: Academic performance influences perceived satisfaction.*

## Data Analysis

The survey conducted has been analysed by using the SPSS software, which provides advanced statistical analysis capabilities. More concretely, Structural Equation Modeling (SEM) (Ringle et al. 2014) and Partial Least Squares (PLS) (Ringle et al. 2014) have been adopted by using SmartPLS to test the associated hypotheses and evaluate the measurement model’s validity recommended by (Hair et al. 2017).

Looking at the demographic characteristics of participants (summary reported in Table 1), in terms of gender there is a fundamental balance as the 52.6% of participants (n = 80) are females and the 47.4% (n = 72) are males. Most participants (54.6%, n = 83) are between 21 and 23 years old, while over 24 are significantly represented (32.2%, n = 49) both with a minority under 21 (13.2%, n = 20). The majority of participants (55.9%, n = 85) are involved in Information Systems majors, with a significant contribution from students in Computer Science (26.3%, n = 40); a minor participation (2%, n = 3) is from Computer Engineering and Networks.

**Table 1:** Demographic Characteristics of the participants

Number of participants (n) = 152			
		**n**	**%**
Gender	*Male*	72	47.4
	*Female*	80	52.6
Age Group	*18–20*	20	13.2
	*21–23*	83	54.6
	*Over 24*	49	32.2
Discipline	*Computer Science*	40	26.3
	*Information Systems*	85	55.9
	*Computer Engineering and Networks*	3	2
	*Other*	24	15.8
Program	*Undergraduate*	138	90.8
	*Postgraduate*	14	9.2
Previous experience with Collaborative Technology	*Less than a year*	14	9.2
	*1–3 years*	50	32.9
	*4 + years*	88	57.9

Additionally, the 90.8% (n = 138) of participants is undergraduate, while the reminder part (9.2%, n = 14) is postgraduate. An interesting statistic is about the experience adopting collaborative technology in education at the survey time. The great majority of participants has reported a previous experience adopting collaborative technology in education. More concretely, the 57.9% of participants has declared more than four years of experience, while the 32.9% 1–3 years; only the 9.2% has less than one year of experience.

The results are presented in detail in the following subsections, which refer to common steps in PLS analysis. Section 5.1 addresses the Measurement Model Analysis, which aims to determine Convergent Validity and Discriminant Validity. The former represents the convergent validity if more measures are used for an individual construct, while the latter defines the extent to which measures of a given construct differ from different constructs’ measurements in the same model (Ab Hamid et al. 2017; Hair et al. 2011, 2012; Hulland 1999). Then, in Sect. 5.2 Structural Model is adopted to check the relationship among research constructs. Structural Model allows to measure each endogenous latent variable (LV’s) Coefficient of Determination (R2). Finally, it provides an evaluation of the path coefficients among LVs to test the hypotheses (Hair et al. 2011; Loehlin and Beaujean 2016; Sharma and Kim 2012; Tenenhaus et al. 2005).

### Measurement model analysis

The values associated with the different metrics adopted in the study are reported in Table 2. More concretely, Average Variance Extracted (AVE), Composite Reliability (CR), and Cronbach’s Alpha (CA) are used to measure Convergent Validity along with the loading of the measurements related to their corresponding constructs (Hulland 1999). AVE refers to the average variance shared between a construct and its related measures, which indicates convergent validity; CR evaluates the internal consistency among scale items, and CA measures the reliability of the construct indicators (Hulland 1999).

The selected metrics have different thresholds to ensure that the obtained measures are reliable and valid for further analysis as reported in (Hair et al. 2011, 2012; Hulland 1999). Typically, a value for AVE that is 0.5 or higher is assumed to be appropriate. For CR and CA values are in the range 0–1. The typical accepted values are in the range 0.7–0.9 and should not be lower than 0.6.

**Table 2:** Measurement Model Analysis

Construct	Item	Loading	AVE	CR	CA
Collaborative Technology (CT)	**CT1**: Enabling collaboration	0.899	0.805	0.925	0.881
	**CT2**: Effective collaboration	0.907			
	**CT3**: Engagement, communication and knowledge development	0.917			
Collaborative Learning (CL)	**CL1**: Knowledge development	0.888	0.832	0.937	0.900
	**CL2**: Brainstorming	0.930			
	**CL3**: Group thinking	0.917			
Effectiveness	**Effectiveness 1**: Develop and document expected outcomes	0.922	0.849	0.918	0.822
	**Effectiveness 2**: Data model and gathering	0.921			
Efficiency	**Efficiency 1**: Time constraints (group)	0.839	0.660	0.885	0.842
	**Efficiency 2**: Individual vs. group tasks	0.752			
	**Efficiency 3**: Difficulty of Individual tasks	0.855			
	**Efficiency 4**: Ideation & brainstorming	0.799			
Enjoyment	**Enjoyment 1**: Collaborative vs. individual work	0.882	0.767	0.868	0.698
	**Enjoyment 2**: Use of technology	0.870			
Academic Performance (SP)	**SP1**: Learning effectiveness	0.919	0.847	0.917	0.819
	**SP2**: Quality of learning	0.921			
Perceived Satisfaction (PS)	**PS 1**: Outcome	0.892	0.826	0.905	0.809
	**PS 2**: Decision making within a group	0.926			

Measured values in Table 2) show that all loadings are higher than the target threshold (0.7). On the other side, AVE measures fall in the range 0.660–0.849, while CR values range from 0.868 to 0.937 and CA measures are above 0.69.

There are different methods to assess Discriminant Validity (Campbell and Fiske 1959; Rönkkö and Cho 2022). In this study we adopt the Fornell and Larcker Criterion (Fornell and Larcker 1981), the Heterotrait-Monotrait Ratio (HTMT) (Henseler et al. 2015), and the Cross-Loading method (Ab Hamid et al. 2017).

According to the Fornell and Larcker Criterion (reported in Table 3), in order to have valid measurements, the diagonal elements in the relevant rows and columns should be much larger than the off-diagonal elements (Fornell and Larcker 1981; Hulland 1999).

**Table 3:** Fornell and Larcker Criterion

	Academic Performance	Collaborative Learning	Collaborative Technology	Effectiveness	Efficiency	Enjoyment	Perceived Satisfaction
Academic Performance	**0.920**						
Collaborative Learning	0.687	**0.912**					
Collaborative Technology	0.811	0.733	**0.897**				
Effectiveness	0.797	0.833	0.813	**0.921**			
Efficiency	0.747	0.567	0.693	0.666	**0.812**		
Enjoyment	0.758	0.735	0.823	0.817	0.683	**0.876**	
Perceived Satisfaction	0.760	0.574	0.685	0.627	0.812	0.659	**0.909**

Heterotrait-Monotrait Ratio (HTMT) is seen as an alternative method with proven high-performance to determine discriminant validity (Henseler et al. 2015). Typical acceptance thresholds for HTMT are between 0.85 (Clark and Watson 1995; Tabri and Elliott 2012) and 0.90 (Gold et al. 2001; Teo et al. 2008). Table 4 reports the measured values that fall within the recommended thresholds. A value higher than the threshold indicates a lack of discriminant validity.

**Table 4:** Discriminant Validity Heterotrait-Monotrait Ratio (HTMT)

	*Academic Performance*	*Collaborative Learning*	*Collaborative Technology*	*Effectiveness*	*Efficiency*	*Enjoyment*	*Perceived Satisfaction*
Academic Performance							
Collaborative Learning	0.800						
Collaborative Technology	0.865	0.822					
Effectiveness	0.812	0.762	0.835				
Efficiency	0.886	0.712	0.791	0.793			
Enjoyment	0.841	0.895	0.675	0.652	0.884		
Perceived Satisfaction	0.557	0.676	0.818	0.769	0.756	0.888	

Cross-loading assessment is often considered as an item-level discriminant validity (Ab Hamid et al. 2017) as the loading indicators on the target factor have to be more significant than on the other constructs. The acceptance threshold is normally 0.70 (Ab Hamid et al. 2017). Table 5 shows the measured Cross-Loading.

**Table 5:** Assessment of Cross-Loading

	*Academic Performance*	*Collaborative Learning*	*Collaborative Technology*	*Effectiveness*	*Efficiency*	*Enjoyment*	*Perceived Satisfaction*
AP 2	**0.919**	0.572	0.632	0.662	0.721	0.612	0.726
AP1	**0.921**	0.691	0.86	0.804	0.654	0.781	0.673
CL 1	0.608	**0.888**	0.646	0.724	0.544	0.599	0.552
CL 2	0.654	**0.930**	0.693	0.758	0.512	0.73	0.511
CL 3	0.615	**0.917**	0.666	0.797	0.497	0.678	0.511
CT 1	0.758	0.708	**0.899**	0.76	0.65	0.683	0.615
CT 2	0.734	0.674	**0.907**	0.772	0.654	0.746	0.624
CT 3	0.69	0.585	**0.886**	0.649	0.557	0.792	0.603
Effectiveness 1	0.703	0.842	0.725	**0.922**	0.765	0.772	0.563
Effectiveness 2	0.766	0.694	0.773	**0.921**	0.721	0.734	0.593
Efficiency 1	0.646	0.561	0.654	0.754	**0.839**	0.631	0.731
Efficiency 2	0.492	0.347	0.447	0.762	**0.752**	0.524	0.549
Efficiency 3	0.514	0.394	0.492	0.731	**0.855**	0.452	0.589
Efficiency 4	0.721	0.493	0.610	0.728	**0.799**	0.582	0.722
Enjoyment 1	0.692	0.756	0.699	0.799	0.637	**0.882**	0.583
Enjoyment 2	0.635	0.527	0.744	0.63	0.559	**0.870**	0.573
PS 1	0.76	0.578	0.649	0.645	0.742	0.608	**0.926**
PS 2	0.61	0.457	0.593	0.483	0.735	0.591	**0.892**

### Structural model

After assessing the validity of measurements, the structural modelling can be conducted as an essential phase of SEM (Tenenhaus et al. 2005). It includes two different steps that deal respectively with the measurement of endogenous LV’s Coefficient of Determination (R^2^) and the evaluation of the path coefficients among (Hair et al. 2011; Sharma and Kim 2012).

Looking at R^2^ three possible levels are normally considered: substantial level refers to values around 0.670, average to values around 0.333, while values below 0.190 are considered to be weak (Chin 1998). The values of R^2^ are recommended to be sufficiently high to ensure that the model has a minimal degree of explanatory power (Sharma and Kim 2012).

**Fig. 2:**
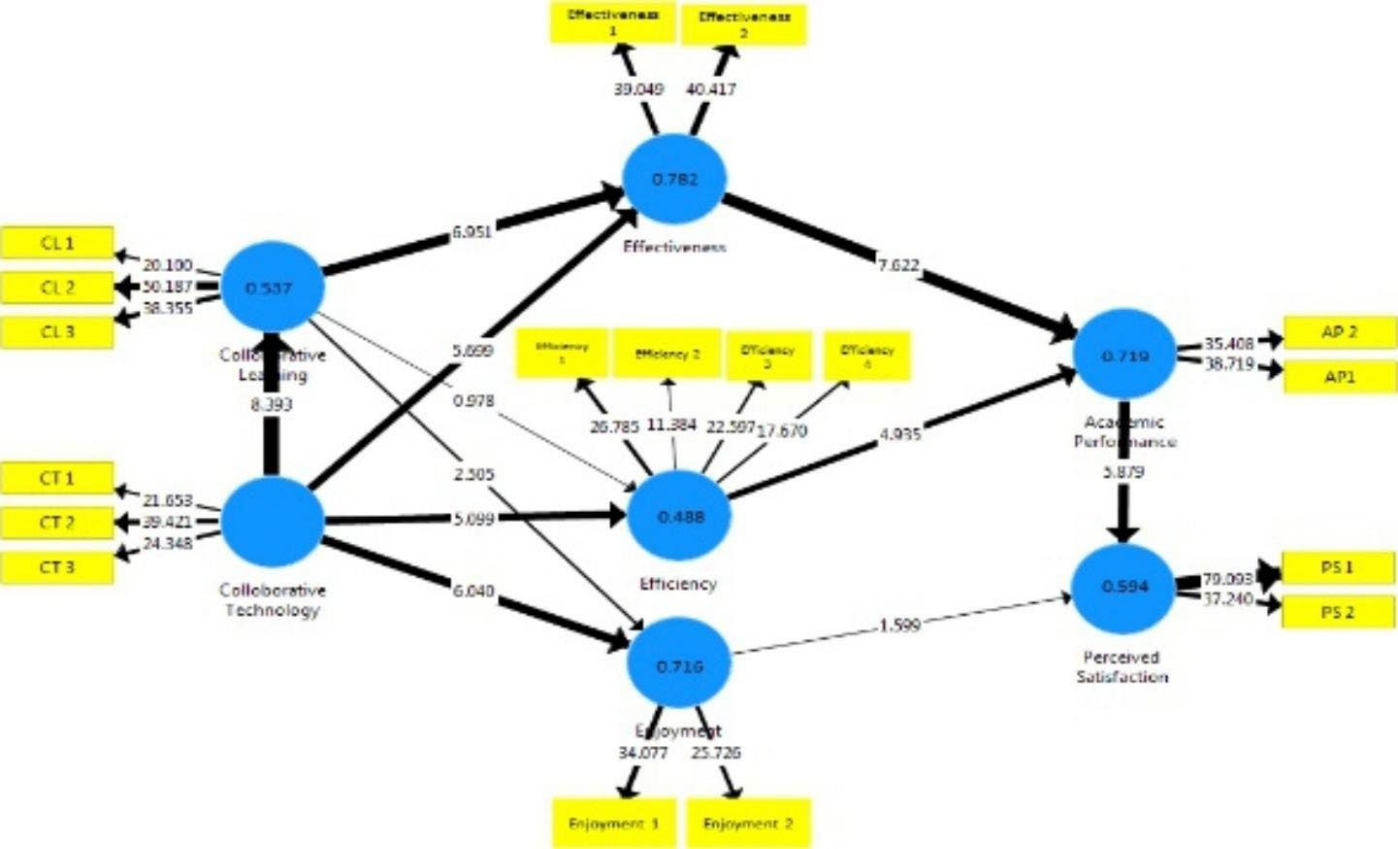
Path Analysis

Figure 2 summarises path analysis and the measured values for R^2^ are reported accordingly. According to such measures, Effectiveness (0.782), Enjoyment (0.716), and Academic Performance (0.719) are well over the substantial level. Collaborative Learning (0.537), Efficiency (0.488) and Perceived Satisfaction (0.594) present values close to the substantial threshold.

The Path-Coefficient is a standardised regression coefficient (ß) in PLS-SEM to test the structural model and hypothesis by highlighting the direct effect among the constructs (Hair et al. 2011). The bootstrap approach in PLS path analysis tests the relevance of path coefficients associated with the standard error of the path and t-value (Hair et al. 2011). Thus, a total of 5000 bootstrap sub-samples have been applied to examine the path coefficients and make the hypotheses assessment.

P-value is adopted to assess the consistency (Hair et al. 2011; Kazár 2014). Path coefficients are considered to be significant if the p-value is lower than 0.05 and not significant if higher than 0.05 (Hair et al. 2011; Kazár 2014). Table 6 provides an overview of conducted measures. According to p-value measurement, H3 and H10 are not significant, while all others fall within the significant range.

**Table 6:** Hypotheses testing result

Hypothesis	Relationship	Std. Beta	Std. Error	t-value	Decision	p-value
H 1	Collaborative Technology → Collaborative Learning	0.726	0.084	8.720	Supported	0.000^**^
H 2	Collaborative Learning → Effectiveness	0.515	0.077	6.698	Supported	0.000^**^
**H 3**	**Collaborative Learning → Efficiency**	**0.138**	**0.136**	**0.935**	**Not Supported**	**0.350** ^**ns**^
H 4	Collaborative Learning → Enjoyment	0.281	0.111	2.560	Supported	0.011*
H 5	Collaborative Technology → Effectiveness	0.435	0.079	5.499	Supported	0.000^**^
H 6	Collaborative Technology → Efficiency	0.592	0.114	5.273	Supported	0.000^**^
H 7	Collaborative Technology → Enjoyment	0.620	0.098	6.271	Supported	0.000^**^
H 8	Effectiveness → Academic Performance	0.534	0.072	7.449	Supported	0.000^**^
H 9	Efficiency → Academic Performance	0.392	0.080	4.847	Supported	0.000^**^
**H 10**	**Enjoyment →** **Perceived Satisfaction**	**0.195**	**0.121**	**1.630**	**Not Supported**	**0.104** ^**ns**^
H 11	Academic Performance → Perceived Satisfaction	0.612	0.105	5.839	Supported	0.000^**^

H1 is definitely significant according to measured values (ß =0.726, t = 8.720, p < 0.01), as well as H2 (ß =0.515, t = 6.698, p < 0.01), H4 (ß =0.281, t = 2.560, p < 0.05), H5 (ß =0.435, t = 5.499, p < 0.01), H6 (ß =0.592, t = 5.273, p < 0.01), H7 (ß =0.620, t = 6.271, p < 0.01), H8 (ß =0.534, t = 7.449, p < 0.01) H9 (ß =0.392, t = 4.847, p < 0.01), H11 (ß =0.612, t = 5.839, p < 0.01). As mentioned before, H3 and H10 are not supported by empirical measurements according to the analysis thresholds.

## Discussion and implications

According to the quantitative analysis conducted, nine out of eleven hypotheses have been accepted. There is no contradiction or significant inconsistency with results obtained in other studies as in literature. For instance, in (Alalwan et al. 2019; Alenazy et al. 2019; Hernández-Sellés et al., 2019), the impact of technology within collaborative learning environments is overall assessed positively, as well as the influence of collaborative technology has resulted in an increased academic performance and perceived satisfaction in (Al-Rahmi et al. 2014; Al-Rahmi and Zeki 2017). Our research conducted in a specific context has reiterated the importance of technology on the establishment of effective collaborative learning environments. Most part of the underlying theory is in line with the analysis of collected data. Assuming the intrinsic complexity of the knowledge building process within collaborative learning environments, the study has pointed out once again the critical role of technology in general terms and its clear impact on the overall quality of experience.

On the other hand, the study doesn’t demonstrate a direct relationship between Collaborative Learning and efficiency. That is in a way surprising although largely understandable in the context proposed where collaboration among students presents some challenges and it’s not always perceived by students as a value but rather like some additional effort that not necessarily leads to efficiency in developing tasks.

We reiterate the lack of direct relationship between enjoyment and perceived satisfaction, which is rather related to performance. In other words, students are still primary concerned about their final result. However, technology seems to positively impact enjoyment.

From a more theoretical perspective, we have combined into unique analysis framework concepts from Bloom’s taxonomy and Nonaka’s theory. Bloom’s taxonomy focuses mostly on learning objectives in educational settings, while Nonaka’s theory addresses knowledge creation within organizational contexts. The process to develop and share knowledge in collaborative learning presents in fact significant similarities and common challenges with a more generic organizational context (Baloian and Zurita 2012; Lee and Schottenfeld 2014).

From a more practical perspective, system design is not an easy task at an educational level and considering this specific task may have affected the study, meaning we do not expect necessarily the same outcome looking at contexts different from system design.

## Conclusions and future work

This study addressed the impact of collaborative technology on the overall quality of experience at different levels of learning. The focus is on the relationship between the knowledge development and quality of experience understood as academic performance and perceived satisfaction within collaborative learning environments that extensively rely on technology.

The analysis conducted on the collected data is in line with suggested hypotheses with two relevant exceptions as extensively discussed in the previous section. In summary, the study has clearly pointed out a direct impact of technology on the collaborative knowledge building process, as well as directly and indirectly on the overall quality of learning experience. Additionally, results show a positive impact of technology along the collaborative knowledge development process and a strong correlation among the different quality of learning experience parameters considered.

Those findings are expected to contribute to the Saudi Arabia’s vision 2030 and, more holistically, to the assessment of collaborative learning environments that extensively rely on technology.

Despite the evident impact of technology on learning performance and experience, we also reiterate the relevance of other aspects related to a human approach to education. It is currently object of research but it is out of the scope of this paper.

As the experiment took place in Saudi Arabia universities looking specifically at programs that address some aspect of system design, considering a variety of contexts and different countries could further consolidate the main findings of the research. Moreover, some of the concepts identified are suitable to support further explorations - i.e. specific studies on the different dimensions along the knowledge building process.

From a more theoretical perspective, the study has not addressed all the dimensions of analysis provided by the Nonaka’s framework; it will be object of future work.

Finally, the study has not explicitly addressed the Technology Acceptance Model (TAM) under the key assumption that collaborative technology is consolidated and largely accepted. It could be an object of further research.
